# Deciphering the effects of polydatin on neuropathic pain and motor performance in a chronic constriction injury model: role of GABA and opioid receptors and neuroprotective properties

**DOI:** 10.3389/fphar.2025.1691130

**Published:** 2025-11-07

**Authors:** Sadaf Abdian, Sajad Fakhri, Amir Kiani, Mohammad Mehdi Gravandi, Fatemeh Abbaszadeh, Mohammad Hosein Farzaei, Ehsan Mohammadi-Noori, Javier Echeverría

**Affiliations:** 1 Student Research Committee, Kermanshah University of Medical Sciences, Kermanshah, Iran; 2 Pharmaceutical Sciences Research Center, Health Institute, Kermanshah University of Medical Sciences, Kermanshah, Iran; 3 Regenerative Medicine Research Center, Kermanshah University of Medical Sciences, Kermanshah, Iran; 4 Neurobiology Research Center, Institute of Neuroscience and Cognition, Shahid Beheshti University of Medical Sciences, Tehran, Iran; 5 Departamento de Ciencias del Ambiente, Facultad de Química y Biología, Universidad de Santiago de Chile, Santiago, Chile

**Keywords:** polydatin, chronic constriction injury, neuropathic pain, oxidative stress, inflammation, opioid receptor, benzodiazepine receptor

## Abstract

**Background:**

Chronic nerve pain is a complex and debilitating condition. Considering the complex pathophysiology of chronic nerve pain, researchers aim to develop novel multi-targeting agents. Polydatin (PLD), a natural multi-targeting compound, has demonstrated antioxidant and anti-inflammatory properties, positioning it as a promising option for alleviating chronic nerve pain.

**Purpose:**

The current study investigated the potential of PLD in treating neuropathic pain induced by chronic constriction injury (CCI) in rats.

**Materials and Methods:**

Sixty male Wistar rats were assigned to ten distinct groups: sham, CCI (negative control), gabapentin (GBP, positive control, 100 mg/kg), and three PLD treatment groups (5, 10, 15 mg/kg). Groups seven to ten received flumazenil (FLU, 0.5 mg/kg) and naloxone (NAL, 0.1 mg/kg) with or without the most potent dose of PLD. Hot plate, acetone drop, inclined plane, and open field tests were used to monitor behavioral changes for 14 days. Biochemical assays were performed to assess changes in catalase (CAT), glutathione (GSH), and nitrite. Additionally, the zymography method was utilized to measure serum levels of matrix metalloproteinase (MMP)-2 and MMP-9 on days 7 and 14. Finally, on day 14, histopathological changes were also assessed.

**Results and Discussion:**

PLD alleviated neuropathic pain and enhanced locomotor activity following CCI. It also increased antioxidant CAT/GSH levels, reducing oxidative nitrite levels, and inflammatory MMP-2 and MMP-9. From the histological results, PLD improved myelin sheaths and protected against axonal swelling, and reduced the dysregulation of gaps in the nerve fibers. FLU and NAL partially reversed these positive effects of PLD.

**Conclusion:**

PLD could be a promising multi-targeting candidate for treating neuropathic pain and motor dysfunction through its anti-inflammatory, antioxidant, and neuroprotective properties, acting on opioid and GABA receptors.

## 1 Introduction

Neuropathic pain is a complex and debilitating condition that severely impacts the individual’s quality of life. It arises from damage or dysfunction in the nervous system, leading to various sensory distortions that challenge normal sensory processing (Szewczyk et al., 2022). The origins of neuropathic pain lie in various medical conditions that induce damage or dysfunction within the nervous system (Feldman et al., 2019). Neuropathic pain could happen in the context of chronic conditions (e.g., diabetes), viruses (e.g., post-herpetic neuralgia) (Hadley et al., 2016), spinal cord injury (SCI) (Shiao and Lee-Kubli, 2018), multiple sclerosis, and stroke through which disrupt the pathways through which pain signals are transmitted and processed (Nurmikko et al., 2010; Mohanan et al., 2023). The neurobiological underpinnings of neuropathic pain involve alterations in both peripheral and central nervous system functions. Changes in nerve signaling can lead to spontaneous pain experiences and the phenomena of hyperalgesia and allodynia. In hyperalgesia, even minor injuries or stimuli can evoke overwhelming pain responses, while allodynia can cause sensations like light touch or temperature changes to be perceived as painful (Jensen and Finnerup, 2014).

Experimental models of peripheral nerve injury enable us to investigate the mechanisms responsible for hypersensitive responses, to discover new analgesic targets (Fleetwood-Walker et al., 2007). Chronic constriction injury (CCI) is a widely utilized experimental model for studying neuropathic pain, primarily in rodent species. This model was first established by Bennett and Xie in 1988 and has since become a standard method for investigating the mechanisms of neuropathic pain and testing potential therapeutic interventions (Bennett and Xie, 1988; Gopalsamy et al., 2019). The CCI model accurately replicates the symptoms of neuropathic pain seen in humans, including allodynia and hyperalgesia, which arise from peripheral nerve damage and inflammation (Bennett and Xie, 1988; Okamoto et al., 2001). One of the key advantages of the CCI model is its ability to induce long-lasting and widespread behavioral changes, making it an essential tool for understanding the underlying mechanisms of chronic pain (Austin et al., 2012). In addition to pain-related symptoms, the CCI model is also associated with various behavioral disabilities, including anxiety and depression, thereby providing a comprehensive framework for evaluating potential therapeutic interventions for neuropathic pain and its comorbid psychological conditions (Fonseca-Rodrigues et al., 2021). Accordingly, the CCI model can serve as a crucial platform for advancing our understanding of neuropathic pain and developing effective treatments.

Oxidative stress plays a significant role in the pathophysiology of pain following nerve damage, particularly through the increased production of reactive oxygen species (ROS). ROS are critical substrates for excitatory nociceptive transmission in the spinal dorsal horn (Nishio et al., 2013). Increased ROS levels lead to neuronal hyperexcitability, which is a hallmark of neuropathic pain conditions (Kim et al., 2015). Increased oxidative stress markers have been observed in animals subjected to CCI, correlating with inflammation. Pro-inflammatory cytokines like interleukin 6 (IL-6) and tumor necrosis factor-alpha (TNF-α) are released after nerve injury. These cytokines can enhance pain perception by activating signaling pathways involving mitogen-activated protein kinase (MAPKs) and nuclear factor-kappa B (NF-κB), leading to heightened neuronal excitability and chronic pain conditions (Popiolek-Barczyk and Mika, 2016; Vanderwall and Milligan, 2019). Matrix metalloproteinase (MMP)-2 and MMP-9 are crucial in modulating the inflammatory response. They facilitate the migration of immune cells such as macrophages and neutrophils into inflamed tissues, which is essential for effective immune responses but can also lead to tissue damage if not properly regulated. For instance, MMP-9 activates pro-inflammatory cytokines, further amplifying the inflammatory response (Nissinen and Kähäri, 2014; Fingleton, 2017). As another pharmacological aspect, benzodiazepine receptors, particularly those associated with the GABA-A receptor, and opioid receptors both play important roles in pain modulation. Their interaction can lead to enhanced analgesic effects, which is an important consideration in pain management (Gear et al., 1997; Primeaux et al., 2006).

Natural products are rich sources of active compounds (Fakhri et al., 2022a; Majnooni et al., 2023), such as polyphenols, especially stilbenoids, which are part of the resveratrol family. Polydatin (PLD, 3,4′,5-trihydroxystilbene-3-β-D-glucoside) is one of the most popular stilbenoids. PLD, commonly referred to as resveratrol-3-β-mono-D-glucoside, is derived from the roots of *Reynoutria japonica* Houtt [Polygonaceae]. It is also present in grapes, peanuts, hop cones, chocolate, cocoa, and a variety of other foods. This compound has a higher absorption than resveratrol, especially from the intestine (Fakhri et al., 2021). PLD is more stable and water-soluble than resveratrol, and it can reduce oxidative stress, inflammation, and cell death (Jiang et al., 2017; Fakhri et al., 2021). We previously showed the neuroprotective effects of PLD in the context of SCI. PLD showed antioxidant and anti-inflammatory effects after SCI. PLD also increased neuronal survival while decreasing lesion size in the spinal cord (Bagheri Bavandpouri et al., 2024). We also unveiled the antinociceptive effect and appropriate doses of PLD passing through the L-arginine/nitric oxide/cyclic GMP/ATP-sensitive potassium channel pathway (Abdian et al., 2025). As another confirmed model of neuropathic pain, CCI was previously employed to evaluate the anti-neuropathic pain effects of active compounds. Accordingly, we previously showed the neuroprotective effects of astaxanthin after CCI in rats (Hashemi et al., 2024).

Considering the lack of effective treatments with lower side effects and high efficacy against CCI, in this study, we aim to investigate the effects of PLD on neuropathic pain and motor impairment resulting from CCI. Mechanistically, we evaluated the antioxidative, anti-inflammatory, and neuroprotective effects of PLD, mediated through opioid and benzodiazepine receptors, which may contribute to alleviating neuropathic pain and improving motor function.

## 2 Materials and methods

### 2.1 Chemicals and reagents

Polydatin (PLD) and gabapentin (GBP) were obtained from Sigma–Aldrich (USA). We also sourced naloxone (NAL), an opioid receptor blocker, from Caspian Tamin Pharmaceutical Company in Iran, and flumazenil (FLU), a GABA receptor antagonist, from Hameln in Germany.

### 2.2 Animals

The experimental procedures were conducted following the ethical guidelines for animal use and care established by the Kermanshah University of Medical Sciences and the National Institutes of Health (IR.KUMS.AEC.1401.019). It should be noted that the animals were housed in standard conditions, featuring a 12-h light and 12-h dark cycle, with a comfortable temperature maintained between 25 °C and 27 °C, and easy access to water and food.

Sixty male Wistar rats, aged 8–10 weeks, were organized into 10 separate groups: sham (surgery with no injury then receiving distilled water as a vehicle), CCI (negative control, received injury then receiving distilled water as a vehicle), gabapentin (GBP, positive control group, received injury then 100 mg/kg of GBP), three treatment groups of PLD (received injury then 5, 10, and 15 mg/kg of PLD), and four additional groups that received FLU at a dose of 0.5 mg/kg and NAL at a dose of 0.1 mg/kg, either alone or in combination with the optimal dose of PLD (10 mg/kg). All substances were administered intraperitoneally (i.p.) daily for 14 days. Distilled water was used as the vehicle for all treatments.

### 2.3 Chronic constriction injury

To induce CCI, the sciatic nerve was partially ligated with sutures, causing persistent compression. This procedure was performed under anesthesia using ketamine and xylazine (80/10 mg/kg, i.p.) (Hashemi et al., 2024). The hair on the lower back and thighs of the rat was shaved, followed by a lateral incision made on the left thigh to expose the underlying tissues. The biceps muscle was then dissected to expose the sciatic nerve, which is typically located in the upper thigh region. The dorsal third of the sciatic nerve was partially ligated using a suture. Four loose chromic catgut ligaments (4.0), with a 1-mm gap between each ligature, were placed around the sciatic nerve (Zamani et al., 2025). This creates a persistent compression that leads to nerve injury (Rezq et al., 2020).

### 2.4 Behavioral test

The rats were assessed for behavioral changes on days 1, 3, 5, 7, 9, 11, 13, and 14 post-surgery.

#### 2.4.1 Acetone drop test

To assess cold sensitivity in rats, acetone was sprayed on their paws, and their pain responses were scored based on a scale from 0 to 4. This scoring system provides a structured way to quantify the intensity of pain reactions: No reaction (0), flinching only (1), partial paw withdrawal between 5 and 30 s (2), extended withdrawal of more than 30 s (3), and severe pain (4) (Fakhri et al., 2018; Sobeh et al., 2020).

#### 2.4.2 Hot plate test

To evaluate heat hyperalgesia and an increased sensitivity to painful heat stimuli, the rats were placed on a hot plate. Rats were placed on a heated surface between 50 °C and 54 °C. The environment was enclosed to prevent escape, ensuring the animal remained on the hot plate during the assessment. The primary outcomes measured were the time it took for the animal to exhibit nocifensive behaviors, such as licking its paw or jumping off the plate. Finally, the paw withdrawal latency (PWL) was recorded considering the cut-off time of 60 s to prevent any potential harm to the animal (Muthuraman and Singh, 2011; Fakhri et al., 2022b).

#### 2.4.3 Open field test

The open-field behavior test measures anxiety, depression, and motor activity in animals. In this study, rats were placed in a black box and their movements were monitored for 5 min. Their behaviors, including rearing, grooming, and crossing, were recorded over 5 minutes (Hashemi et al., 2024). Crossing serves as a direct measure of locomotor activity. A higher number of crossings, rearing, and grooming indicates lower levels of pain sensation, as it suggests a greater willingness to explore the environment (Suarez and Gallup, 1981; Sturman et al., 2018).

#### 2.4.4 Inclined plane test

This test evaluates the ability of animals to maintain their balance on a sloped surface, providing insights into their motor capabilities and neurological health. The inclined plane consisted of a flat surface that could be adjusted at angles between 0 and 60°. Rats were placed on the inclined plane, and the steepest angle at which they could remain upright for at least 5 s was recorded (Chang et al., 2008).

### 2.5 Biochemical test

After the behavioral tests were completed, the rats were euthanized, and their blood was collected for biochemical analysis.

#### 2.5.1 Glutathione and catalase assay

To evaluate changes in glutathione (GSH) and catalase (CAT) levels, which indicate antioxidant status, we employed a colorimetric assay. GSH levels were determined using the Ellman method, which measures the decline of GSH in the presence of Ellman’s reagent (5,5-dithio-bis-(2-nitrobenzoic acid, DTNB). For this assay, 60 μL of rat serum and 100 μL of 2 mg/mL DTNB were mixed in a plate, and phosphate buffer (50 μL) was added. After 10 min of incubation at 37 °C, absorbance was read at 412 nm (Eyer and Podhradský, 1986). CAT activity was measured using the Aebi method, which involves assessing changes in serum hydrogen peroxide (H_2_O_2_) levels. In this assay, 20 μL of serum was mixed with 100 μL of H_2_O_2_ (65 mM) and incubated for 4 min. The reaction was stopped using ammonium molybdate, and the concentration of H_2_O_2_ was read at 405 nm. Changes in concentration were reported as a percentage of the control (Aebi, 1984; Fakhri et al., 2022b).

#### 2.5.2 Nitrite assay

Nitrite levels in serum samples were measured using the Griess assay. Serum (100 µL) was mixed with sulfanilamide solution (50 µL) and incubated in the dark for 5 min. After that, naphthyl ethylene diamine dihydrochloride (NEDD; 50 µL) was added to the mixture, and it was incubated for 30 min. Optical density was measured at 540 nm (Sun et al., 2003).

### 2.6 Zymography

MMP-2 and MMP-9 activities were measured on days 7 and 14. The serum samples containing 100 μg of total protein were loaded onto sodium dodecyl sulfate polyacrylamide gel electrophoresis (SDS-PAGE) with 0.1% gelatin, followed by electrophoresis at 150 V. After a 1-h wash in Triton X-100 buffer, the gel was incubated at 37 °C for 18 h in a calcium-containing incubation buffer. The gel was then stained with Coomassie blue and destained with acetic acid and methanol. Clear bands indicated MMPs activities, which were quantified using ImageJ software (Fakhri et al., 2022b).

### 2.7 Histological analysis

On the 14th day, the ligated sciatic nerve segments were dissected and preserved in formalin (10%). Then, serial sections prepared from paraffin-embedded tissue were stained with hematoxylin and eosin (H&E). These sections were qualitatively assessed under a light microscope at 400× magnification to evaluate axonal degeneration using standard histological methods (Muthuraman et al., 2008; Bakare and Owoyele, 2020). The percentage of damaged area was quantified using ImageJ (NIH).

### 2.8 Statistical analysis

Results were analyzed using version 8.0 of Prism software. One-way ANOVA was followed by Tukey’s multiple comparison test, and two-way ANOVA was followed by Bonferroni correction to adjust for multiple comparisons. The area under the curve was determined using the trapezoidal method. Data are expressed as mean ± standard error of the mean (SEM), with significance defined at *p* < 0.05. The effect sizes were calculated using eta squared (ηp^2^).

## 3 Results

### 3.1 Behavioral result

#### 3.1.1 Acetone drop test

**FIGURE 1:**
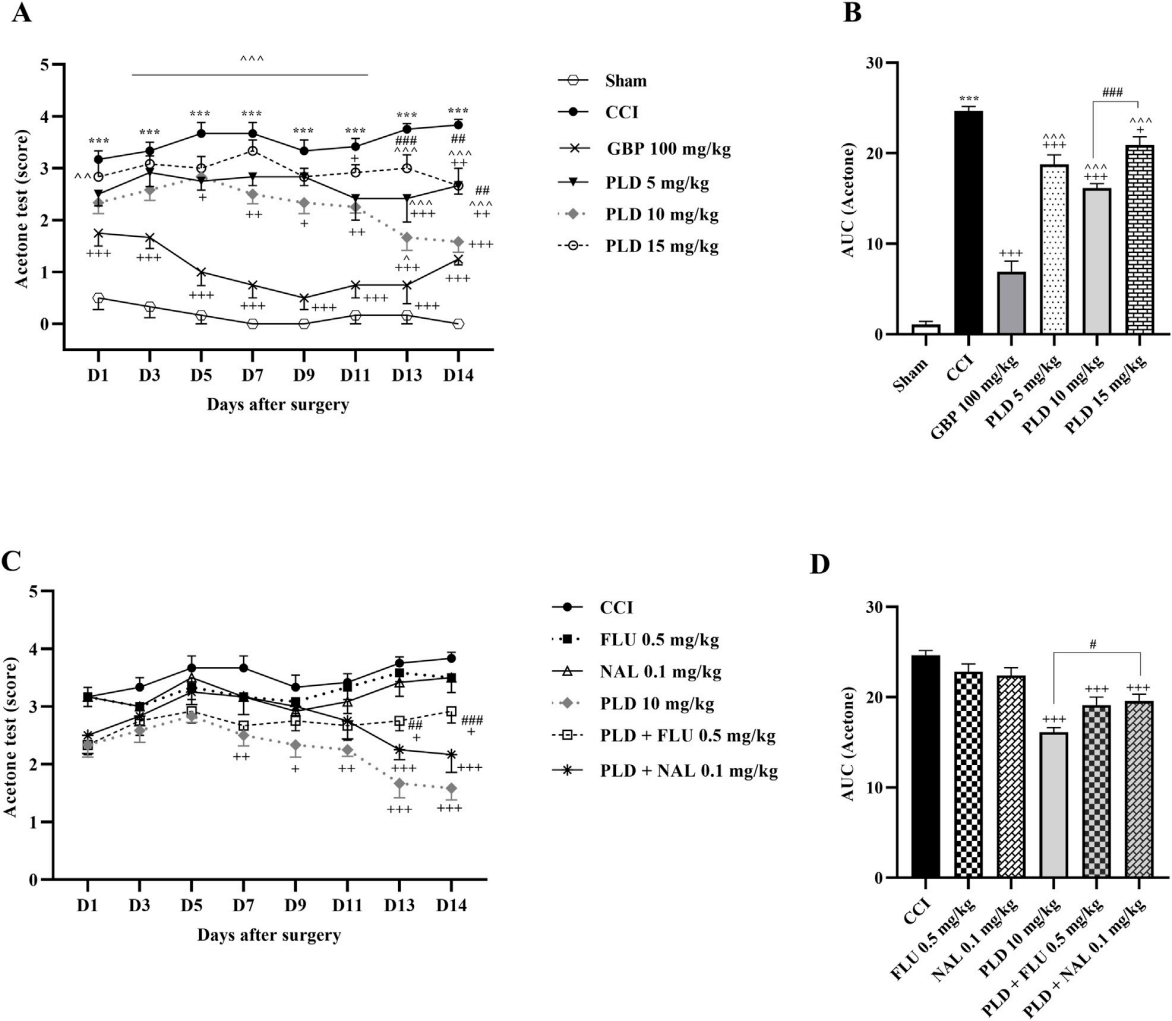
The impact of PLD on cold allodynia in rats following the CCI model. Two-way ANOVA revealed that CCI significantly reduced the tolerance threshold of cold stimulus, and PLD was able to reverse it **(A)**, and this result was confirmed by AUC analysis **(B)**. While FLU and NAL, when administered alone, did not affect cold allodynia, their combined use with PLD mitigated the effects of PLD **(C)**, a result that was confirmed by AUC analysis **(D)**. The data are shown as mean ± SEM. ^***^ *p* < 0.001 vs. sham; ^+^ *p* < 0.05, ^++^ *p* < 0.01, ^+++^ *p* < 0.001 vs. CCI; ^*p* < 0.05, ^^*p* < 0.01, ^^^*p* < 0.001 vs. GBP; ^#^ *p* < 0.05, ^##^ *p* < 0.01, ^###^ *p* < 0.001 vs. PLD (10 mg/kg). CCI, Chronic constriction injury; GBP, Gabapentin; FLU, Flumazenil; NAL, Naloxone; PLD, Polydatin.

The acetone drop test demonstrated a significant enhancement in the paw withdrawal reflex for the CCI group when compared to the sham group (*p* < 0.001) (Figure 1A). Furthermore, treatment with PLD at three different doses, similar to GBP (as a positive control), improved the tolerance to cold stimuli compared to the CCI group (*p* < 0.05, effect size = 0.76). Among the three doses of PLD, the most pronounced effect was observed in the group treated with PLD at a dose of 10 mg/kg, starting from day 5 (*p* < 0.05) (Figure 1A). Additionally, the area under the curve for cold allodynia in these groups showed a significant decrease compared to the CCI group (*p* < 0.05) (Figure 1B). In contrast, the paw withdrawal reflex in response to cold stimuli in rats treated with FLU or NAL alone did not differ significantly from the CCI group (Figure 1C). However, the data indicated that administering NAL thirty minutes before PLD (10 mg/kg) significantly diminished the analgesic effects of PLD (*p* < 0.01), resulting in an enhancement in cold allodynia in this group (Figure 1C). Moreover, there was a notable increase in the area under the curve for cold allodynia in this group in comparison to the PLD (10 mg/kg) treated group (*p* < 0.05) (Figure 1D).

#### 3.1.2 Heat hyperalgesia

**FIGURE 2:**
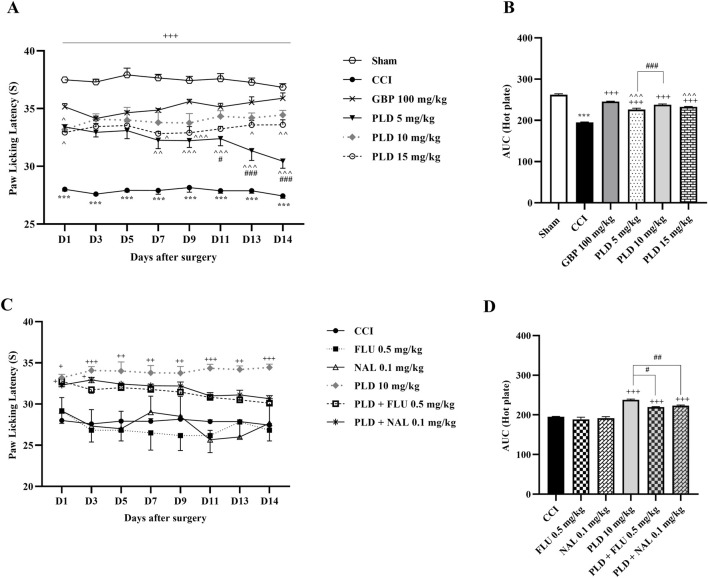
The impact of PLD on thermal hyperalgesia in rats following the CCI model. Two-way ANOVA revealed that CCI significantly reduced paw-licking latency during thermal hyperalgesia, and PLD was able to reverse it **(A)**, and this result was confirmed by AUC analysis **(B)**. While FLU and NAL, when administered alone, did not affect thermal hyperalgesia, their combined use with PLD mitigated the effects of PLD **(C)**, a result that was confirmed by AUC analysis **(D)**. The data are shown as mean ± SEM. ^***^ *p* < 0.001 vs. sham; ^+^ *p* < 0.05, ^++^ *p* < 0.01, ^+++^ *p* < 0.001 vs. CCI; ^*p* < 0.05, ^^*p* < 0.01, ^^^*p* < 0.001 vs. GBP; ^#^ *p* < 0.05, ^##^ *p* < 0.01, ^###^ *p* < 0.001 vs. PLD (10 mg/kg). CCI, Chronic constriction injury; GBP, Gabapentin; FLU, Flumazenil; NAL, Naloxone; PLD, Polydatin.

The hot plate test showed that the sham group had consistent PWL, while the CCI group reacted strongly to heat, significantly lowering PWL from the first day (*p* < 0.001) (Figure 2A). Treatment with the three doses of PLD, similar to GBP, increased PWL after CCI (*p* < 0.001, effect size = 0.93) (Figure 2A). The area under the curve clearly showed an increase in these groups compared to the CCI group (*p* < 0.001) (Figure 2B). There was no difference in PWL between the 10 mg/kg PLD and GBP groups (Figure 2B), and the 10 mg/kg PLD dose proved to be more effective than the 5 mg/kg PLD dose (*p* < 0.001). Rats treated with FLU or NAL showed no significant change in PWL compared to the CCI group. Additionally, co-administering PLD with FLU or NAL significantly reduced PWL compared to PLD alone, indicating increased thermal hyperalgesia due to receptor blockade (*p* < 0.05 and *p* < 0.01, respectively) (Figures 2C,D).

#### 3.1.3 Open field test

**FIGURE 3:**
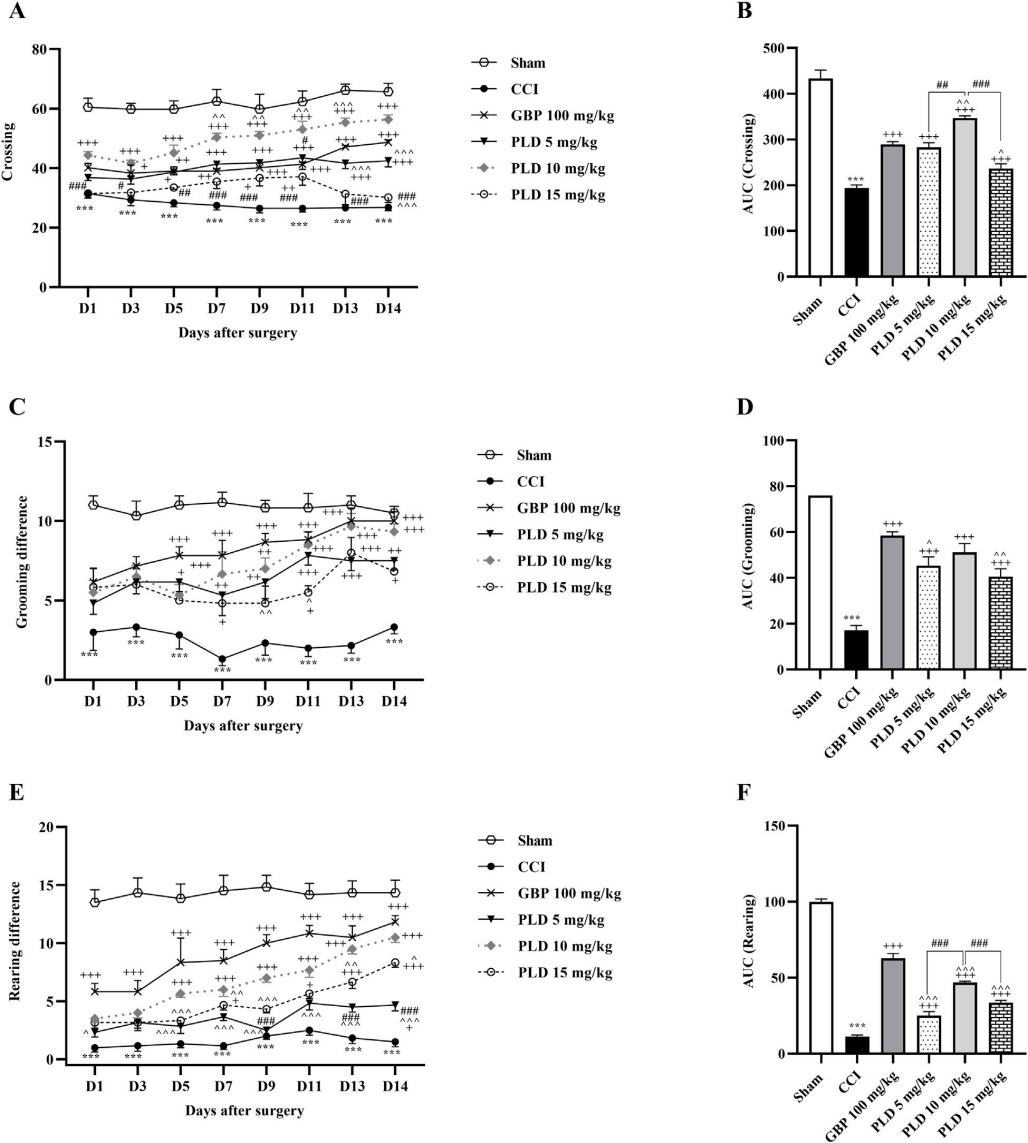
The impact of PLD on locomotor activity in rats following the CCI model. Two-way ANOVA revealed that CCI significantly reduced crossing **(A)**, grooming **(C)**, and rearing **(E)**, and PLD was able to reverse them, and these results were confirmed by AUC analysis **(B,D,F)**. The data are shown as mean ± SEM. ^***^ *p* < 0.001 vs. sham; ^+^ *p* < 0.05, ^++^ *p* < 0.01, ^+++^ *p* < 0.001 vs. CCI; ^*p* < 0.05, ^^*p* < 0.01, ^^^*p* < 0.001 vs. GBP; ^#^ *p* < 0.05, ^##^ *p* < 0.01, ^###^ *p* < 0.001 vs. PLD (10 mg/kg). CCI, Chronic constriction injury; GBP, Gabapentin; PLD, Polydatin.

The open field test results revealed a significant decrease in all three components of locomotor activity, crossing (Figure 3A), grooming (Figure 3C), and rearing (Figure 3E), between CCI and sham groups (*p* < 0.001). PLD administration, similar to GBP, enhanced these motor function parameters, crossing (*p* < 0.05, effect size = 0.90), grooming (*p* < 0.05, effect size = 0.74), and rearing (*p* < 0.05, effect size = 0.93), in comparison to the CCI group. The area under the curve most clearly showed the reduction of these parameters following the CCI model and the positive effect of PLD on them (*p* < 0.001) (Figures 3B,D,F). The group that received a dose of 10 mg/kg had the most significant effect among the three doses of PLD administered (*p* < 0.01).

**FIGURE 4:**
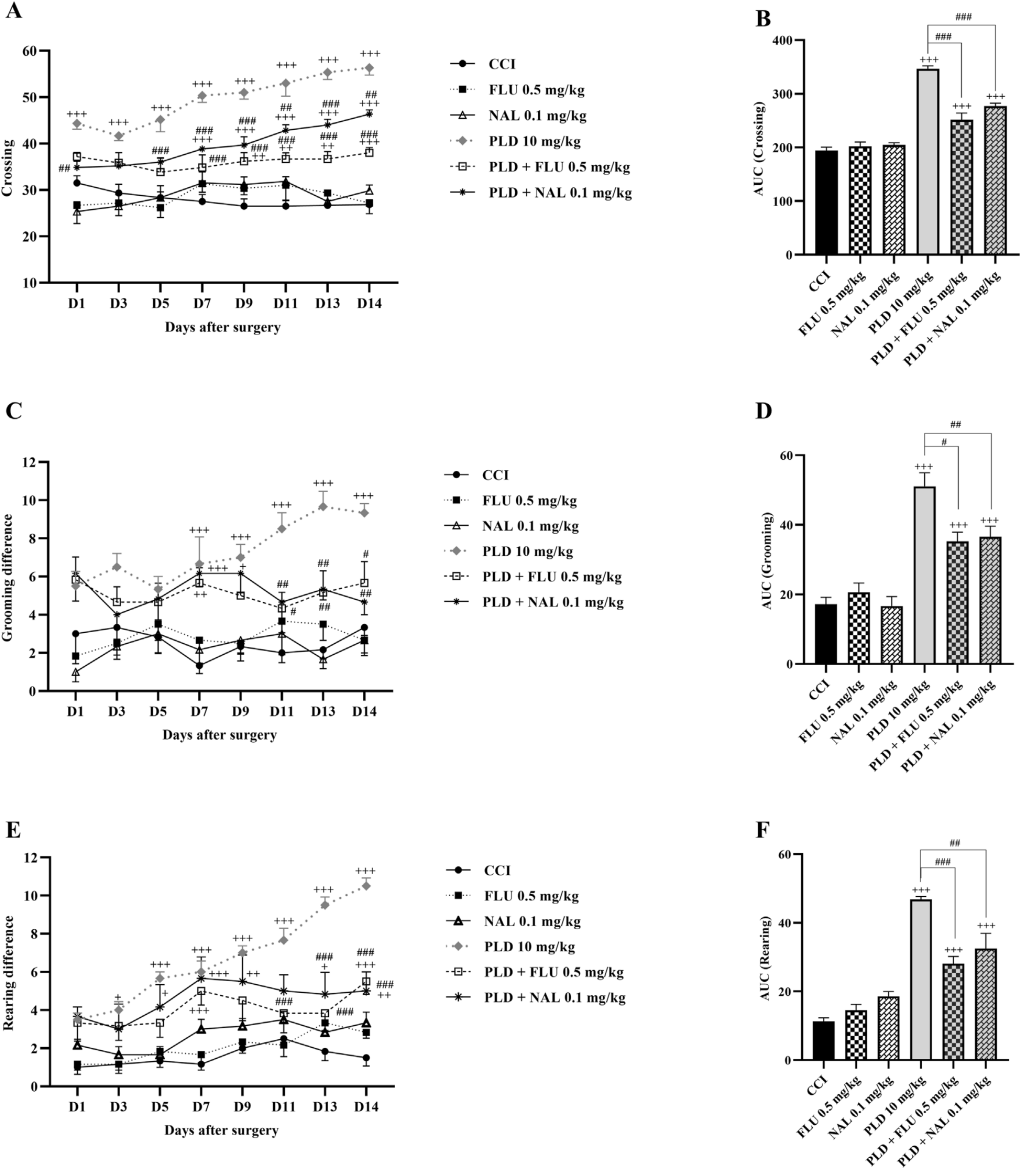
The impact of co-administering PLD with FLU and NAL on locomotor activity in rats following the CCI model. FLU and NAL, when administered alone, did not affect crossing **(A)**, grooming **(C)**, and rearing **(E)**; their combined use with PLD mitigated the effects of PLD, and these results were confirmed by AUC analysis **(B,D,F)**. The data are shown as mean ± SEM. ^+^ *p* < 0.05, ^++^ *p* < 0.01, ^+++^ *p* < 0.001 vs. CCI; ^#^ *p* < 0.05, ^##^ *p* < 0.01, ^###^ *p* < 0.001 vs. PLD (10 mg/kg). CCI, Chronic constriction injury; GBP, Gabapentin; FLU, Flumazenil; NAL, Naloxone; PLD, Polydatin.

FLU and NAL alone did not affect locomotor activity (Figures 4A,C,E). Nevertheless, the FLU and NAL groups exhibited a statistically significant reduction in these parameters compared to the 10 mg/kg PLD group (*p* < 0.05). These changes were clearly illustrated by the area under the curve (*p* < 0.05) (Figures 4B,D,F).

#### 3.1.4 Inclined plane test

**FIGURE 5:**
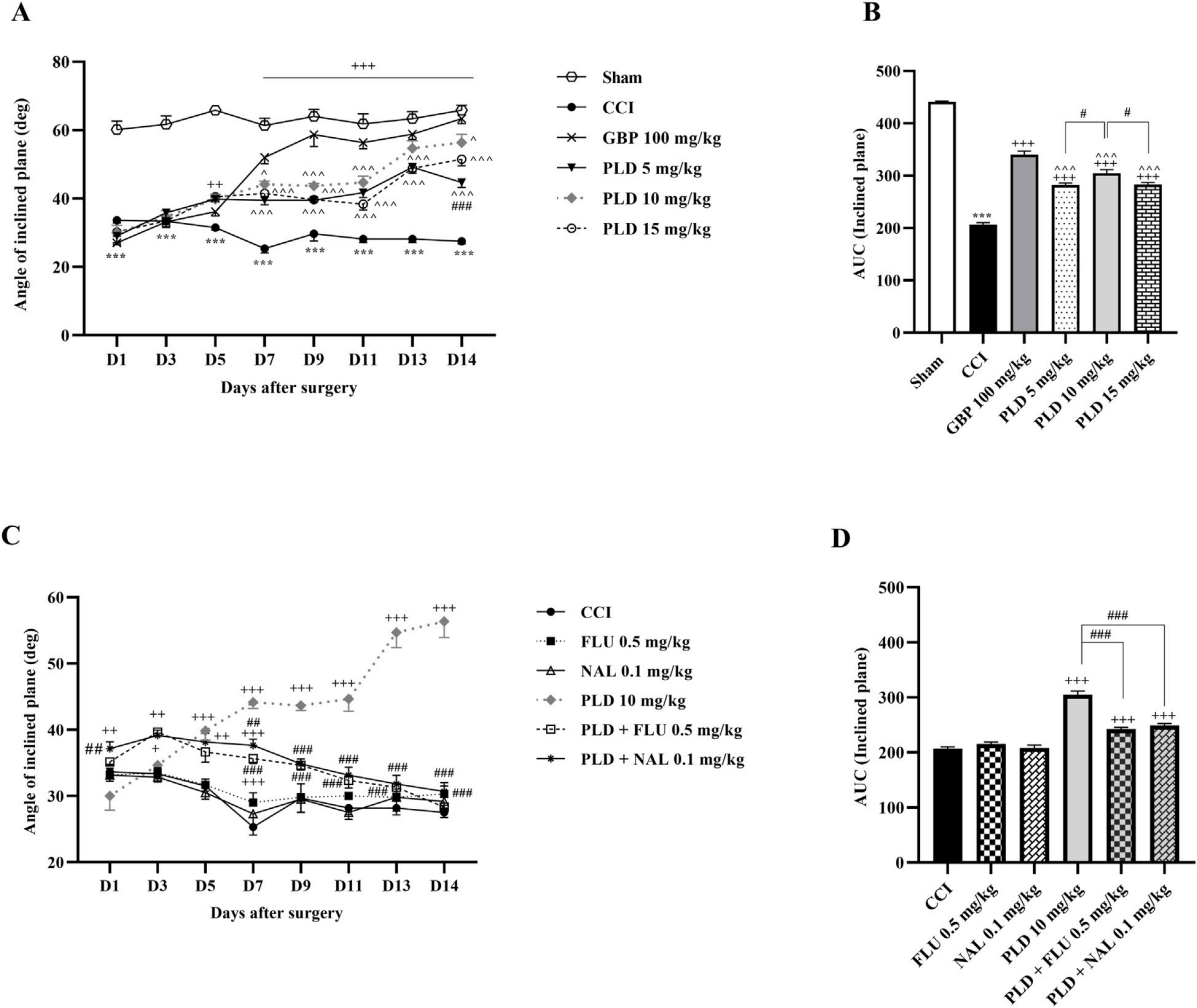
The impact of PLD on joint function and mobility in rats following the CCI model. Two-way ANOVA revealed that CCI significantly reduced joint function and mobility, and PLD was able to reverse it **(A)**, and this result was confirmed by AUC analysis **(B)**. While FLU and NAL, when administered alone, did not affect joint function and mobility, their combined use with PLD mitigated the effects of PLD **(C)**, a result that was confirmed by AUC analysis **(D)**. The data are shown as mean ± SEM. ^***^ *p* < 0.001 vs. sham; ^+^ *p* < 0.05, ^++^ *p* < 0.01, ^+++^ *p* < 0.001 vs. CCI; ^*p* < 0.05, ^^*p* < 0.01, ^^^*p* < 0.001 vs. GBP; ^#^ *p* < 0.05, ^##^ *p* < 0.01, ^###^ *p* < 0.001 vs. PLD (10 mg/kg). CCI, Chronic constriction injury; GBP, Gabapentin; FLU, Flumazenil; NAL, Naloxone; PLD, Polydatin.

The results indicated that rats in the CCI group exhibited a significant decline in their joint function and ability to remain on the ramps compared to the sham group (*p* < 0.01). Treatment with the three doses of PLD positively impacted the rats’ functional recovery after the CCI (*p* < 0.001, effect size = 0.93) (Figure 5A). The area under the curve in these groups showed a significant enhancement compared to the CCI group (*p* < 0.001) (Figure 5B). Of the three doses of PLD administered, the best effect was observed in the group treated with 10 mg/kg of PLD (*p* < 0.05) (Figure 5B).

Two additional treatments, FLU and NAL, did not lead to significant improvements compared to the CCI group. When FLU and NAL were administered alongside PLD, there was a significant decrease in performance compared to the group receiving only PLD (10 mg/kg) (*p* < 0.01). The area under the curve showed this reduction more prominently (*p* < 0.001).

### 3.2 Biochemical analysis

#### 3.2.1 Glutathione/catalase assay

**FIGURE 6:**
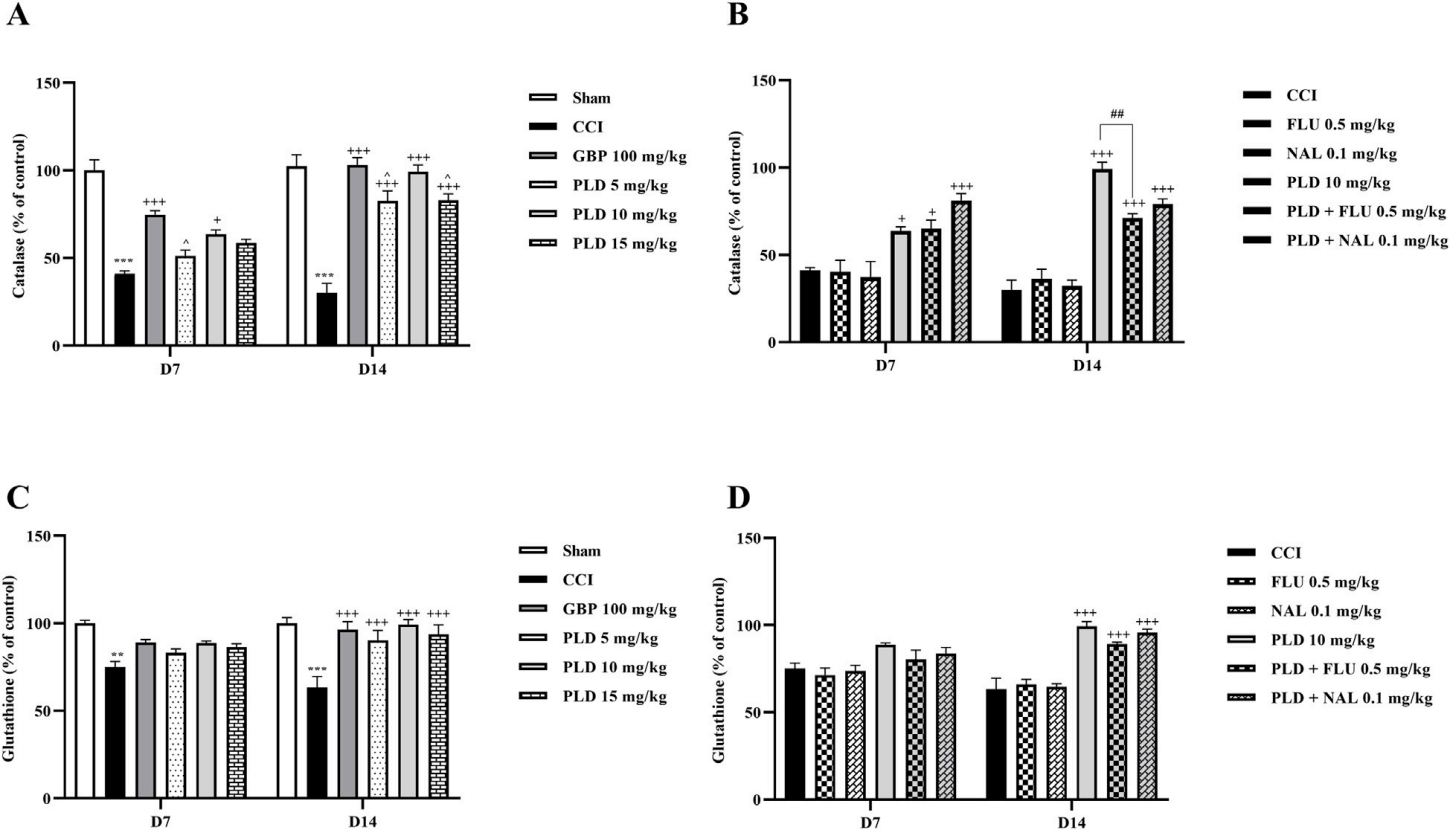
The impact of PLD on CAT and GSH activity in rats following the CCI model. Two-way ANOVA revealed that CCI significantly reduced CAT **(A)** and GSH **(C)** activity, and PLD reversed these effects. While FLU and NAL, when administered alone, did not affect CAT **(B)** and GSH **(D)** activity, their combined use with PLD partially mitigated the effects of PLD. The data are shown as mean ± SEM. ^**^ *p* < 0.01, ^***^ *p* < 0.001 vs. sham; ^+^ *p* < 0.05, ^+++^ *p* < 0.001 vs. CCI; ^^^ *p* < 0.05, vs. GBP; ^##^ *p* < 0.01 vs. PLD (10 mg/kg). CAT, Catalase; CCI, Chronic constriction injury; GBP, Gabapentin; GSH, Glutathione; FLU, Flumazenil; NAL, Naloxone; PLD, Polydatin.

The biochemical assay results on days 7 and 14 showed that serum CAT (*p* < 0.001) (Figure 6A) and GSH (*p* < 0.01) (Figure 6C) levels in the CCI group rats were meaningfully lower than those in the sham group. Administration of three doses of PLD significantly increased these CAT (*p* < 0.05) and GSH (*p* < 0.001) levels compared to the CCI group. In these regards, PLD 10 mg/kg had an effect almost similar to GBP. Among the three doses of PLD, the group receiving 10 mg/kg exhibited the most significant effects (*p* < 0.05).

Administration of FLU and NAL separately did not affect serum CAT or GSH levels in rats. However, when they were administered in combination with PLD, they decreased the positive effect of PLD on CAT (Figure 6B) and GSH (Figure 6D) levels. This reduction in effect was significant for CAT (*p* < 0.01) (Figure 6B).

#### 3.2.2 Nitrite assay

**FIGURE 7:**
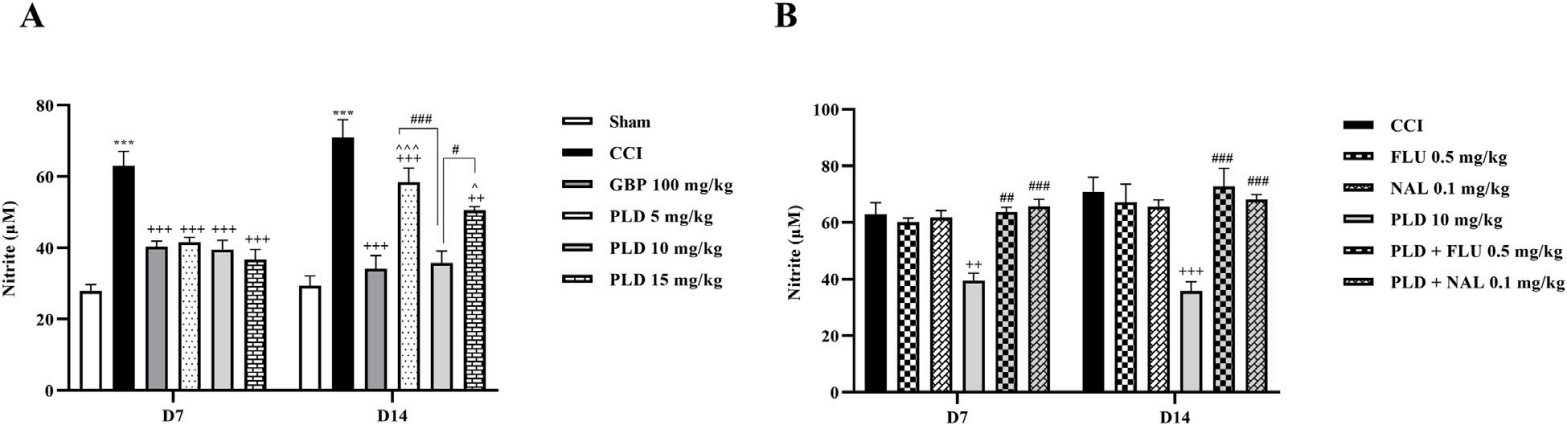
The impact of PLD on the nitrite level in rats following the CCI model. Two-way ANOVA revealed that CCI significantly increased nitrite level, and PLD was able to reverse it **(A)**. While FLU and NAL, when administered alone, did not affect nitrite level **(B)**, their combined use with PLD partially mitigated the effects of PLD. The data are shown as mean ± SEM. ^***^ *p* < 0.001 vs. sham; ^++^ *p* < 0.01, ^+++^ *p* < 0.001 vs. CCI; ^*p* < 0.05, ^^^*p* < 0.001 vs. GBP; ^#^ *p* < 0.05, ^##^ *p* < 0.01, ^###^ *p* < 0.001 vs. PLD (10 mg/kg). CCI, Chronic constriction injury; GBP, Gabapentin; FLU, Flumazenil; NAL, Naloxone; PLD, Polydatin.

The results showed that on days 7 and 14 after surgery, serum nitrite levels in the CCI group were significantly higher than in the sham group (*p* < 0.001). In contrast, the serum nitrite level in all three PLD groups and the GBP group showed a significant decrease compared to the CCI group (*p* < 0.05). Among the three doses of PLD, the 10 mg/kg group showed stronger effects (*p* < 0.05). No significant differences were observed between the PLD 10 mg/kg group and the GBP group (Figure 7A). The administration of FLU and NAL alone did not alter serum nitrite levels in the CCI rats. However, when FLU and NAL were co-administered with PLD, they resulted in significant changes in serum nitrite levels compared to the PLD 10 mg/kg group on days 7 and 14 (*p* < 0.01) (Figure 7B).

### 3.3 Zymography

**FIGURE 8:**
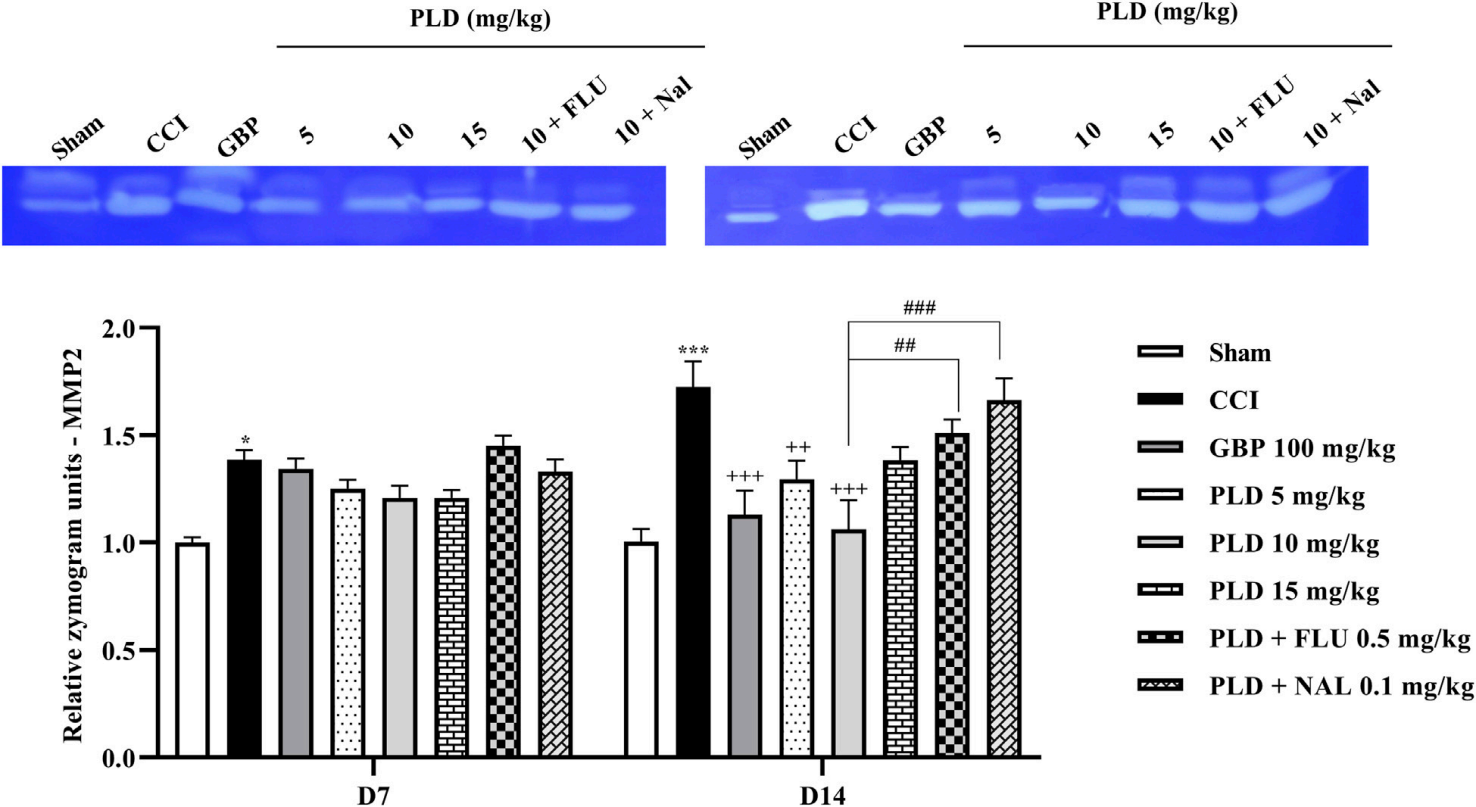
The impact of PLD on the MMP-9 level in rats following the CCI model. Two-way ANOVA revealed that CCI significantly increased the MMP-9 level, and PLD reversed this effect. FLU and NAL mitigated the effects of PLD. The data are shown as mean ± SEM. ^**^ *p* < 0.01, ^***^ *p* < 0.001 vs. sham; ^+^ *p* < 0.05, ^++^ *p* < 0.01 vs. CCI; ^#^ *p* < 0.05, ^##^ *p* < 0.01 vs. PLD (10 mg/kg). CCI, Chronic constriction injury; GBP, Gabapentin; FLU, Flumazenil; MMP, Matrix metalloproteinases; NAL, Naloxone; PLD, Polydatin.

**FIGURE 9:**
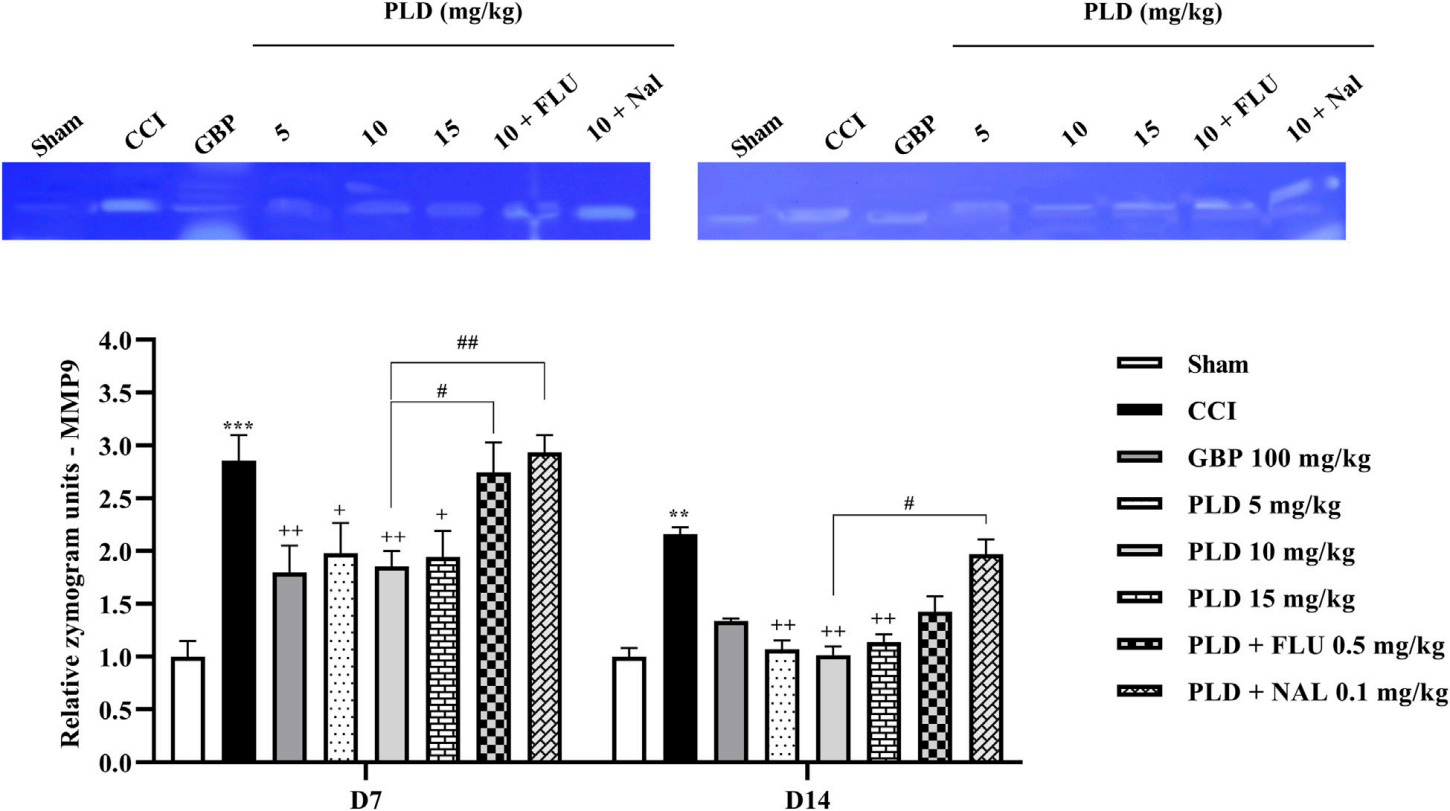
The impact of PLD on the MMP-2 level in rats following the CCI model. Two-way ANOVA revealed that CCI significantly increased the MMP-2 level, and PLD reversed this effect. FLU and NAL mitigated the effects of PLD. The data are shown as mean ± SEM. ^*^ *p* < 0.05, ^***^ *p* < 0.001 vs. sham; ^++^ *p* < 0.01, ^+++^ *p* < 0.001 vs. CCI, ^##^ *p* < 0.01, ^###^ *p* < 0.001 vs. PLD (10 mg/kg). CCI, Chronic constriction injury; GBP, Gabapentin; MMP, Matrix metalloproteinase; FLU, Flumazenil; NAL, Naloxone; PLD, Polydatin.

Zymography results indicated that the CCI model led to a marked increase in MMP-9 (*p* < 0.01) (Figure 8) and MMP-2 (*p* < 0.05) (Figure 9) levels when compared to the sham group on days 7 and 14. PLD treatment doses, particularly in a 10 mg/kg dose, effectively countered this elevation, showing no significant difference from the positive control, GBP (*p* < 0.05). However, administering FLU and NAL 30 min before the injection of PLD 10 mg/kg significantly reduced its beneficial effects on MMP-9 (*p* < 0.05) and MMP-2 (*p* < 0.01) activity levels.

### 3.4 Histological analysis

**FIGURE 10:**
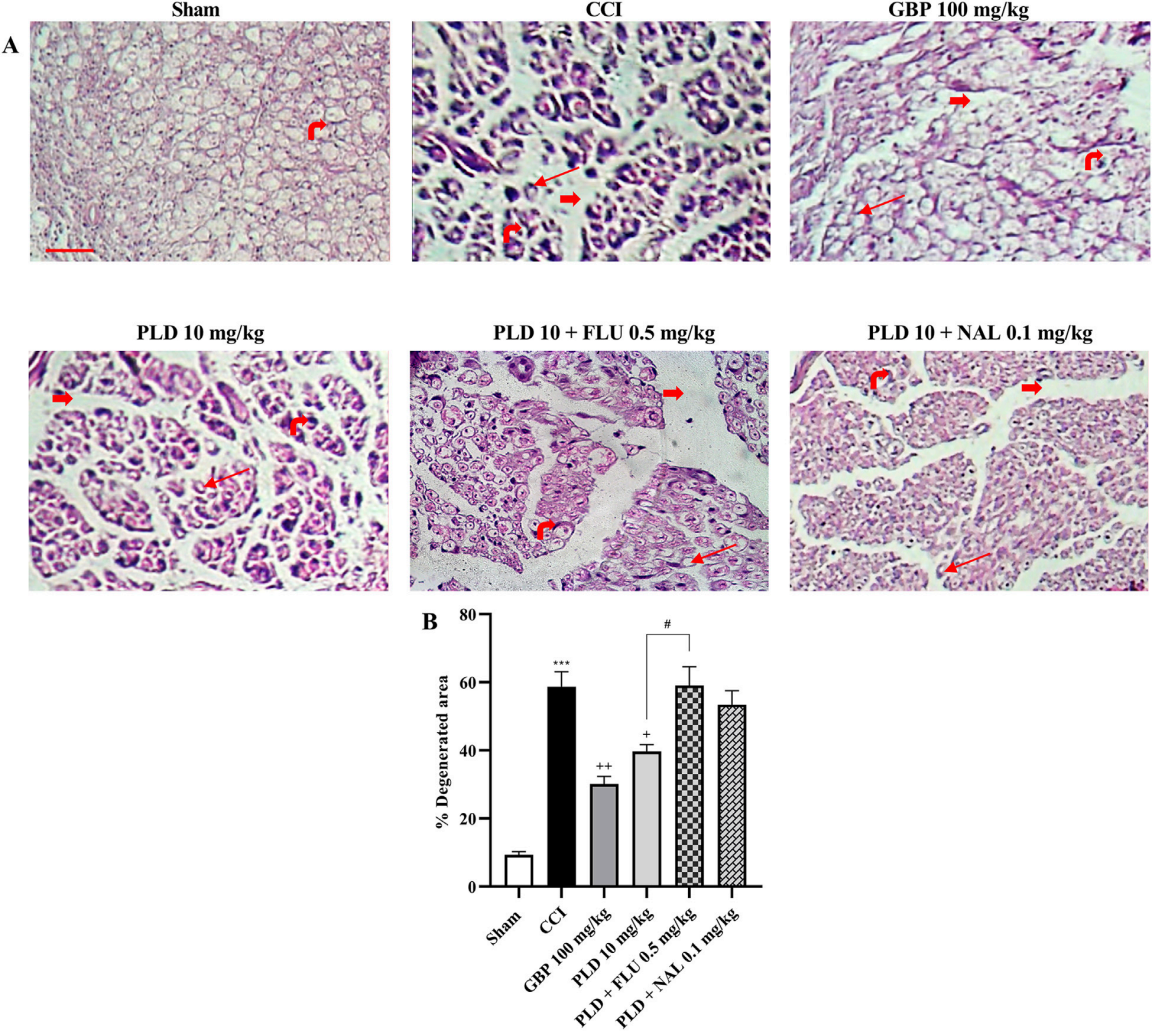
Histological examination of hematoxylin and eosin (H&E) stained sciatic nerve in rats after CCI. Sections of the sciatic nerve were stained using H&E (40x magnification) **(A)**. Thick red arrows indicate gaps between neurons, highlighting significant separations between the nerve fibers. Thin red arrows represent areas of axonal swelling. The rightmost red arrow identifies the nucleus of a Schwann cell. The percentage of damaged area was quantified using ImageJ **(B)**. The data are shown as mean ± SEM. ^***^ *p* < 0.001 vs. sham; ^+^ *p* < 0.05, ^++^ *p* < 0.01, vs. CCI; ^#^ *p* < 0.05 vs. PLD (10 mg/kg). CCI, Chronic constriction injury; GBP, Gabapentin; FLU, Flumazenil; NAL, Naloxone; PLD, Polydatin.

In the sham group, the sciatic nerve sections exhibited well-organized myelin sheaths with only mild hemorrhage present between them. Conversely, the stained nerve sections from the CCI group exhibited disrupted myelin sheaths, marked axonal swelling, and notable gaps between the nerve fibers. Notably, the sections of the GBP (100 mg/kg) and PLD (10 mg/kg) groups demonstrated marked improvement. In contrast, groups co-administered with PLD (10 mg/kg) along with NAL (0.1 mg/kg) or FLU (0.5 mg/kg) reduced the effect of PLD 10 mg/kg (Figure 10A). The CCI group exhibited a significant increase in nerve degeneration compared to the Sham group (*p* < 0.001), confirming successful induction of neuropathic injury. Treatment with GBP (100 mg/kg) markedly reduced the degenerated area compared to the CCI group (*p* < 0.01). Similarly, PLD (10 mg/kg) significantly decreased degeneration (*p* < 0.05), though its effect was less pronounced than GBP. However, co-administration of PLD with FLU (0.5 mg/kg) or NAL (0.1 mg/kg) partially reversed the protective effect of PLD (*p* < 0.05) (Figure 10B).

## 4 Discussion

**FIGURE 11:**
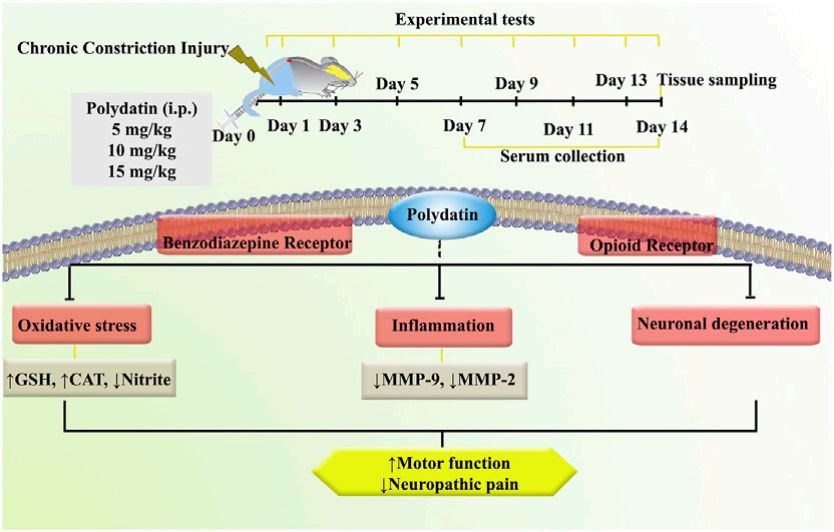
An overview of the study protocol. Polydatin attenuated inflammation, oxidative stress, and neuronal degeneration following CCI. CAT, Catalase; CCI, Chronic constriction injury; GSH, Glutathione; MMP, Matrix metalloproteinase.

The present study investigated the effects of PLD on neuropathic pain in a rat model of CCI. The results demonstrated that PLD treatment significantly attenuated pain-related behaviors and enhanced locomotor activity in the affected rats. Among the tested doses, the 10 mg/kg i.p. administration of PLD elicited the most substantial anti-neuropathic response. Furthermore, PLD administration was associated with a marked reduction in oxidative stress markers, including nitrite, as well as antioxidative enzymes such as CAT and GSH. Besides, PLD increased the levels of inflammatory MMP-2 and MMP-9. From another mechanistic view, the co-administration of FLU, a selective antagonist of benzodiazepine receptors, and NAL, an opioid receptor antagonist, effectively reversed the aforementioned effects of PLD. These findings reveal that such therapeutic effects of PLD pass through opioid and GABA receptors. Our histopathological evaluation confirmed that PLD regulated axonal swelling, myelin sheaths, and pronounced hemorrhage (Figure 11).

The CCI model is a prominent experimental method for studying neuropathic pain, particularly affecting the sciatic nerve in rodents. Following CCI, there is an increase in pro-inflammatory cytokines and chemokines, which activate immune cells and trigger inflammatory responses, contributing to pain hypersensitivity (Safieh-Garabedian et al., 2019; Gopalsamy et al., 2019). This immune activation is linked to heightened oxidative stress, resulting in neuronal damage and increased pain perception (Tan et al., 2009; Komirishetty et al., 2016). Upregulation of oxidant-generating enzymes like nicotinamide adenine dinucleotide phosphate (NADPH) oxidase elevates ROS, causing cellular damage and perpetuating inflammation (Sun et al., 2021a; Olufunmilayo et al., 2023).

Antioxidative defenses are often weakened in CCI models, with reductions in critical antioxidants such as GSH and CAT (Tan et al., 2009; Chen et al., 2022). Additionally, oxidative stress can activate MMPs (Baba and Bhatnagar, 2018), which further exacerbate neuronal damage and pain (Ji et al., 2009). Interventions targeting oxidative stress and neuroinflammation may alleviate neuropathic pain in these models. PLD, a natural derivative of resveratrol, enhances the body’s antioxidant defenses primarily through the activation of the transcription factor nuclear factor erythroid 2 p45-related factor 2 (Nrf2) pathway, which leads to increased expression of antioxidant enzymes such as superoxide dismutase (SOD) and heme oxygenase-1 (HO-1) (Fakhri et al., 2021; Petrikonis et al., 2024). This mechanism helps to mitigate oxidative stress by scavenging ROS and reducing lipid peroxidation (Karami et al., 2022). On the other hand, the anti-inflammatory action of PLD is mediated through several pathways. It inhibits the NF-κB signaling pathway, which is crucial for the expression of pro-inflammatory cytokines like TNF-α, and ILs (Ye et al., 2017; Hu et al., 2022; Ren et al., 2025). By decreasing phospholipase A2 (PLA2) activity and modulating AMPK/Sirt1 signaling, PLD effectively reduces inflammation in various experimental models, including those simulating neuroinflammatory conditions (Chen and Lan, 2017; Sun et al., 2021b). In the present study, consistent with the findings of previous research, we observed that PLD, particularly at a dosage of 10 mg/kg, effectively reduced oxidative stress and MMPs activities. Behaviorally, these beneficial effects were associated with a decrease in neuropathic pain and an enhancement in motor function.

In prior studies, we demonstrated that PLD effectively alleviated neuropathic pain and restored motor function in rats following SCI. This beneficial effect was attributed to its anti-inflammatory and antioxidant properties, which contribute to mitigating the impact of the injury (Bagheri Bavandpouri et al., 2024). In addition, recent advances in the study of natural stilbenoids have highlighted their multifaceted roles in alleviating neuropathic pain and neuroinflammation through diverse molecular mechanisms. Saadabadi et al. (2025) demonstrated that these compounds exert analgesic effects partly via modulation of the TRPA1 ion channel, a critical mediator in pain signal transduction, suggesting a novel target for neuropathic pain treatment (Saadabadi et al., 2025). Additionally, stilbenoids have been shown to stabilize and reduce inflammatory responses by inhibiting key pathways such as phosphoinositide 3-kinase (PI3K)/Akt and NF-κB, resulting in decreased production of pro-inflammatory cytokines, including IL-6, monocyte chemoattractant protein 1 (MCP- 1), and TNF-α (Al-Khayri et al., 2023). PLD’s ability to enhance antioxidative enzymes and shift the MMPs balance toward anti-inflammatory types mirrors the broader neuroprotective profile of stilbenoids reported in recent literature. Importantly, these compounds have also demonstrated neuroprotective effects on neuronal structures, supporting axonal integrity and myelin sheath preservation, which correlates with the histopathological findings of decreased axonal swelling and improved myelin integrity seen in this study. Also, our previous findings revealed that PLD exerts neuroprotection after SCI by preserving neuronal populations within the dorsal and ventral horns and by limiting secondary tissue degeneration in the spinal cord (Bagheri Bavandpouri et al., 2024). The reduction in the activity of MMPs, especially MMP-9, plays a vital role in maintaining the extracellular matrix (ECM) and blood-nerve barrier integrity, which are crucial for normal nerve function (Marcianò et al., 2023). Excessive MMP activities lead to degradation of ECM components and disruption of the blood-nerve barrier, increasing permeability and facilitating infiltration of inflammatory cells, which exacerbates demyelination and axonal injury. MMPs not only participate in tissue remodeling by releasing proteases that degrade ECM components but also induce intracellular protein remodeling by acting on intracellular targets, both of which contribute to the pathogenesis of chronic pain (Dai et al., 2024).

Moreover, PLD exhibited anxiolytic effects in a chronic pain mouse model, highlighting its potential to alleviate not only pain but also the associated anxiety often experienced by individuals with chronic pain conditions (Guan et al., 2020). Wang et al. reported that PLD effectively suppressed depression- and anxiety-like behaviors in a mouse model by inhibiting neuroinflammation and oxidative stress (Wang et al., 2023).

Mechanistically, benzodiazepine receptors are primarily associated with GABA, an inhibitory neurotransmitter that plays a crucial role in calming the nervous system. When benzodiazepines bind to the GABA-A receptor, they enhance the effects of GABA, leading to increased inhibition of neuronal activity. This can result in a reduction of anxiety, muscle relaxation, and also, importantly, a decrease in the perception of pain (Griffin et al., 2013; Zeilhofer et al., 2015). On the other hand, opioid receptors, which include mu, delta, and kappa types, are primarily activated by endogenous peptides (like endorphins) and exogenous compounds (like morphine). Activation of these receptors leads to pain relief (analgesia), as they modulate pain signals in the brain and spinal cord (Corder et al., 2018). These two receptor systems can interact with one another. Research has shown that when benzodiazepine receptors are activated, they can enhance the analgesic effects of opioids (Gear et al., 1997; Lewis et al., 2012). Our research indicated that the beneficial effects of PLD could be decreased by administering FLU or NAL, which suggests that PLD may act through this mechanism.

In addressing the potential limitations of our study, we acknowledge that the sample size may limit the generalizability of our findings. While our results demonstrate significant effects of PLD on neuropathic pain and motor impairment in the CCI model, a larger sample size could provide more robust statistical power and enhance the reliability of our conclusions. However, animal ethics in the use of laboratory animals prefer minimally acceptable numbers *in vivo*. Additionally, the long-term effects of PLD treatment were not assessed in this study. Long-term toxicity studies need to be developed in future studies. Future research should investigate the sustained efficacy of PLD and its impact on neuropathic pain and related behaviors over extended periods. This would provide valuable insights into the potential for PLD as a long-term therapeutic option and help to establish a clearer understanding of its safety profile and therapeutic window in chronic pain management. Potential differences in receptor expression between rodents and humans urge the need to develop well-controlled clinical trials.

## 5 Conclusion

In conclusion, PLD exerts significant antioxidant, anti-inflammatory, and neuroprotective effects that collectively alleviate neuropathic pain and improve motor performance in rats subjected to CCI. The observed modulation of oxidative stress markers, MMPs activities, and histopathological features such as axonal swelling and myelin integrity underscores its neuroprotective capacity. Importantly, the reversal of PLD’s effects by opioid and benzodiazepine receptor antagonists suggests a receptor-mediated mechanism that parallels established pathways in human pain modulation.

These findings highlight the translational potential of PLD as a promising candidate for the development of novel therapeutics targeting neuropathic pain and its comorbidities, such as anxiety and motor dysfunction. Further preclinical and clinical investigations are warranted to determine optimal dosing, long-term efficacy, and safety, paving the way for potential clinical application of PLD in chronic pain management.

## Data Availability

The raw data supporting the conclusions of this article will be made available by the authors, without undue reservation.

## References

[B1] Abdian S. Fakhri S. Abbaszadeh F. Gravandi M. M. Farzaei M. H. Kiani A. (2025). Polydatin relieves nociceptive pain: possible role of L-arginine/NO/cGMP/KATP channel signaling pathway in mice. Jundishapur J. Nat. Pharm. Prod. 20. 10.5812/jjnpp-163665

[B2] Aebi H. (1984). Catalase *in vitro* . Methods Enzym. 105, 121–126. 10.1016/S0076-6879(84)05016-3 6727660

[B3] Al-Khayri J. M. Mascarenhas R. Harish H. M. Gowda Y. Lakshmaiah V. V. Nagella P. (2023). Stilbenes, a versatile class of natural metabolites for inflammation—an overview. Molecules 28, 3786. 10.3390/molecules28093786 37175197 PMC10180133

[B4] Austin P. J. Wu A. Moalem-Taylor G. (2012). Chronic constriction of the sciatic nerve and pain hypersensitivity testing in rats. J. Vis. Exp. 3393, 3393. 10.3791/3393 22433911 PMC3399467

[B5] Baba S. P. Bhatnagar A. (2018). Role of thiols in oxidative stress. Curr. Opin. Toxicol. 7, 133–139. 10.1016/j.cotox.2018.03.005 30338308 PMC6188637

[B6] Bagheri Bavandpouri F. S. Azizi A. Abbaszadeh F. Kiani A. Farzaei M. H. Mohammadi-Noori E. (2024). Polydatin attenuated neuropathic pain and motor dysfunction following spinal cord injury in rats by employing its anti-inflammatory and antioxidant effects. Front. Pharmacol. 15, 1452989. 10.3389/fphar.2024.1452989 39193334 PMC11347411

[B7] Bakare A. O. Owoyele B. V. (2020). Antinociceptive and neuroprotective effects of bromelain in chronic constriction injury-induced neuropathic pain in wistar rats. Korean J. Pain 33, 13–22. 10.3344/kjp.2020.33.1.13 31888313 PMC6944371

[B8] Bennett G. J. Xie Y.-K. (1988). A peripheral mononeuropathy in rat that produces disorders of pain sensation like those seen in man. Pain 33, 87–107. 10.1016/0304-3959(88)90209-6 2837713

[B9] Chang M.-W. Young M.-S. Lin M.-T. (2008). An inclined plane system with microcontroller to determine limb motor function of laboratory animals. J. Neurosci. Methods 168, 186–194. 10.1016/j.jneumeth.2007.09.013 17953994

[B10] Chen L. Lan Z. (2017). Polydatin attenuates potassium oxonate-induced hyperuricemia and kidney inflammation by inhibiting NF-κB/NLRP3 inflammasome activation *via* the AMPK/SIRT1 pathway. Food Funct. 8, 1785–1792. 10.1039/c6fo01561a 28428988

[B11] Chen S.-M. Wang M.-H. Soung H.-S. Tseng H.-C. Fang C.-H. Lin Y.-W. (2022). Neuroprotective effect of l-theanine in a rat model of chronic constriction injury of sciatic nerve-induced neuropathic pain. J. Formos. Med. Assoc. 121, 802–814. 10.1016/j.jfma.2021.08.023 34531102

[B12] Corder G. Castro D. C. Bruchas M. R. Scherrer G. (2018). Endogenous and exogenous opioids in pain. Annu. Rev. Neurosci. 41, 453–473. 10.1146/annurev-neuro-080317-061522 29852083 PMC6428583

[B13] Dai X.-Y. Liu L. Song F.-H. Gao S.-J. Wu J.-Y. Li D.-Y. (2024). Matrix metalloproteinases as attractive therapeutic targets for chronic pain: a narrative review. Int. J. Biol. Macromol. 261, 129619. 10.1016/j.ijbiomac.2024.129619 38272407

[B14] Eyer P. Podhradský D. (1986). Evaluation of the micromethod for determination of glutathione using enzymatic cycling and Ellman’s reagent. Anal. Biochem. 153, 57–66. 10.1016/0003-2697(86)90061-8 3963383

[B15] Fakhri S. Dargahi L. Abbaszadeh F. Jorjani M. (2018). Astaxanthin attenuates neuroinflammation contributed to the neuropathic pain and motor dysfunction following compression spinal cord injury. Brain Res. Bull. 143, 217–224. 10.1016/j.brainresbull.2018.09.011 30243665

[B16] Fakhri S. Gravandi M. M. Abdian S. Akkol E. K. Farzaei M. H. Sobarzo-Sánchez E. (2021). The neuroprotective role of polydatin: neuropharmacological mechanisms, molecular targets, therapeutic potentials, and clinical perspective. Molecules 26, 5985. 10.3390/molecules26195985 34641529 PMC8513080

[B17] Fakhri S. Piri S. Moradi S. Z. Khan H. (2022a). Phytochemicals targeting oxidative stress, interconnected neuroinflammatory, and neuroapoptotic pathways following radiation. Curr. Neuropharmacol. 20, 836–856. 10.2174/1570159X19666210809103346 34370636 PMC9881105

[B18] Fakhri S. Sabouri S. Kiani A. Farzaei M. H. Rashidi K. Mohammadi-Farani A. (2022b). Intrathecal administration of naringenin improves motor dysfunction and neuropathic pain following compression spinal cord injury in rats: relevance to its antioxidant and anti-inflammatory activities. Korean J. Pain 35, 291–302. 10.3344/kjp.2022.35.3.291 35768984 PMC9251389

[B19] Feldman E. L. Callaghan B. C. Pop-Busui R. Zochodne D. W. Wright D. E. Bennett D. L. (2019). Diabetic neuropathy. Nat. Rev. Dis. Prim. 5, 41. 10.1038/s41572-019-0092-1 31197153

[B20] Fingleton B. (2017). Matrix metalloproteinases as regulators of inflammatory processes. Biochim. Biophys. acta. Mol. Cell Res. 1864, 2036–2042. 10.1016/j.bbamcr.2017.05.010 28502592

[B21] Fleetwood-Walker S. M. Proudfoot C. W. J. Garry E. M. Allchorne A. Vinuela-Fernandez I. Mitchell R. (2007). Cold comfort pharm. Trends Pharmacol. Sci. 28, 621–628. 10.1016/j.tips.2007.10.007 17996956

[B22] Fonseca-Rodrigues D. Amorim D. Almeida A. Pinto-Ribeiro F. (2021). Emotional and cognitive impairments in the peripheral nerve chronic constriction injury model (CCI) of neuropathic pain: a systematic review. Behav. Brain Res. 399, 113008. 10.1016/j.bbr.2020.113008 33171146

[B23] Gear R. W. Miaskowski C. Heller P. H. Paul S. M. Gordon N. C. Levine J. D. (1997). Benzodiazepine mediated antagonism of opioid analgesia. Pain 71, 25–29. 10.1016/S0304-3959(97)03332-0 9200170

[B24] Gopalsamy B. Sambasevam Y. Zulazmi N. A. Chia J. S. M. Omar Farouk A. A. Sulaiman M. R. (2019). Experimental characterization of the chronic constriction injury-induced neuropathic pain model in mice. Neurochem. Res. 44, 2123–2138. 10.1007/s11064-019-02850-0 31376053

[B25] Griffin C. E. Kaye A. M. Bueno F. R. Kaye A. D. (2013). Benzodiazepine pharmacology and central nervous system-mediated effects. Ochsner J. 13, 214–223. Available online at: http://www.ncbi.nlm.nih.gov/pubmed/23789008. 23789008 PMC3684331

[B26] Guan S.-Y. Zhang K. Wang X.-S. Yang L. Feng B. Tian D.-D. (2020). Anxiolytic effects of polydatin through the blockade of neuroinflammation in a chronic pain mouse model. Mol. Pain 16, 1744806919900717. 10.1177/1744806919900717 31964240 PMC6977205

[B27] Hadley G. R. Gayle J. A. Ripoll J. Jones M. R. Argoff C. E. Kaye R. J. (2016). Post-herpetic neuralgia: a review. Curr. Pain Headache Rep. 20, 17. 10.1007/s11916-016-0548-x 26879875

[B28] Hashemi B. Fakhri S. Kiani A. Abbaszadeh F. Miraghaee S. Mohammadi M. (2024). Anti-neuropathic effects of astaxanthin in a rat model of chronic constriction injury: passing through opioid/benzodiazepine receptors and relevance to its antioxidant and anti-inflammatory effects. Front. Pharmacol. 15, 1467788. 10.3389/fphar.2024.1467788 39654618 PMC11625551

[B29] Hu L. Luo D. Zhang H. He L. (2022). Polydatin inhibits IL-1β-mediated chondrocyte inflammation and ameliorates cartilage degradation: involvement of the NF-κB and Wnt/β-catenin pathways. Tissue Cell 78, 101865. 10.1016/j.tice.2022.101865 35994920

[B30] Jensen T. S. Finnerup N. B. (2014). Allodynia and hyperalgesia in neuropathic pain: clinical manifestations and mechanisms. Lancet Neurol. 13, 924–935. 10.1016/S1474-4422(14)70102-4 25142459

[B31] Ji R.-R. Xu Z.-Z. Wang X. Lo E. H. (2009). Matrix metalloprotease regulation of neuropathic pain. Trends Pharmacol. Sci. 30, 336–340. 10.1016/j.tips.2009.04.002 19523695 PMC2706286

[B32] Jiang K.-F. Zhao G. Deng G.-Z. Wu H.-C. Yin N.-N. Chen X.-Y. (2017). Polydatin ameliorates staphylococcus aureus-induced mastitis in mice *via* inhibiting TLR2-mediated activation of the p38 MAPK/NF-κB pathway. Acta Pharmacol. Sin. 38, 211–222. 10.1038/aps.2016.123 27890916 PMC5309755

[B33] Karami A. Fakhri S. Kooshki L. Khan H. (2022). Polydatin: pharmacological mechanisms, therapeutic targets, biological activities, and health benefits. Molecules 27, 6474. 10.3390/molecules27196474 36235012 PMC9572446

[B34] Kim H. Y. Lee I. Chun S. W. Kim H. K. (2015). Reactive oxygen species donors increase the responsiveness of dorsal horn neurons and induce mechanical hyperalgesia in rats. Neural Plast. 2015, 293423. 10.1155/2015/293423 26457204 PMC4592728

[B35] Komirishetty P. Areti A. Sistla R. Kumar A. (2016). Morin mitigates chronic constriction injury (CCI)-induced peripheral neuropathy by inhibiting oxidative stress induced PARP over-activation and neuroinflammation. Neurochem. Res. 41, 2029–2042. 10.1007/s11064-016-1914-0 27084773

[B36] Lewis S. S. Loram L. C. Hutchinson M. R. Li C.-M. Zhang Y. Maier S. F. (2012). (+)-Naloxone, an opioid-inactive toll-like receptor 4 signaling inhibitor, reverses multiple models of chronic neuropathic pain in rats. J. Pain 13, 498–506. 10.1016/j.jpain.2012.02.005 22520687 PMC3348259

[B37] Majnooni M. B. Fakhri S. Ghanadian S. M. Bahrami G. Mansouri K. Iranpanah A. (2023). Inhibiting angiogenesis by anti-cancer saponins: from phytochemistry to cellular signaling pathways. Metabolites 13, 323. 10.3390/metabo13030323 36984763 PMC10052344

[B38] Marcianò G. Vocca C. Rania V. Citraro R. De Sarro G. Gallelli L. (2023). Metalloproteases in pain generation and persistence: a possible target? Biomolecules 13, 268. 10.3390/biom13020268 36830637 PMC9953417

[B39] Mohanan A. T. Nithya S. Nomier Y. Hassan D. A. Jali A. M. Qadri M. (2023). Stroke-induced central pain: overview of the mechanisms, management, and emerging targets of central post-stroke pain. Pharm. (Basel) 16, 1103. 10.3390/ph16081103 37631018 PMC10459894

[B40] Muthuraman A. Singh N. (2011). Attenuating effect of Acorus calamus extract in chronic constriction injury induced neuropathic pain in rats: an evidence of anti-oxidative, anti-inflammatory, neuroprotective and calcium inhibitory effects. BMC Complement. Altern. Med. 11, 24. 10.1186/1472-6882-11-24 21426568 PMC3072356

[B41] Muthuraman A. Diwan V. Jaggi A. S. Singh N. Singh D. (2008). Ameliorative effects of Ocimum sanctum in sciatic nerve transection-induced neuropathy in rats. J. Ethnopharmacol. 120, 56–62. 10.1016/j.jep.2008.07.049 18762236

[B42] Nishio N. Taniguchi W. Sugimura Y. K. Takiguchi N. Yamanaka M. Kiyoyuki Y. (2013). Reactive oxygen species enhance excitatory synaptic transmission in rat spinal dorsal horn neurons by activating TRPA1 and TRPV1 channels. Neuroscience 247, 201–212. 10.1016/j.neuroscience.2013.05.023 23707800

[B43] Nissinen L. Kähäri V.-M. (2014). Matrix metalloproteinases in inflammation. Biochim. Biophys. Acta 1840, 2571–2580. 10.1016/j.bbagen.2014.03.007 24631662

[B44] Nurmikko T. J. Gupta S. Maclver K. (2010). Multiple sclerosis-related central pain disorders. Curr. Pain Headache Rep. 14, 189–195. 10.1007/s11916-010-0108-8 20425191

[B45] Okamoto K. Martin D. P. Schmelzer J. D. Mitsui Y. Low P. A. (2001). Pro- and anti-inflammatory cytokine gene expression in rat sciatic nerve chronic constriction injury model of neuropathic pain. Exp. Neurol. 169, 386–391. 10.1006/exnr.2001.7677 11358451

[B46] Olufunmilayo E. O. Gerke-Duncan M. B. Holsinger R. M. D. (2023). Oxidative stress and antioxidants in neurodegenerative disorders. Antioxidants 12, 517. 10.3390/antiox12020517 36830075 PMC9952099

[B47] Petrikonis K. Bernatoniene J. Kopustinskiene D. M. Casale R. Davinelli S. Saso L. (2024). The antinociceptive role of Nrf2 in neuropathic pain: from mechanisms to clinical perspectives. Pharmaceutics 16, 1068. 10.3390/pharmaceutics16081068 39204413 PMC11358986

[B48] Popiolek-Barczyk K. Mika J. (2016). Targeting the microglial signaling pathways: new insights in the modulation of neuropathic pain. Curr. Med. Chem. 23, 2908–2928. 10.2174/0929867323666160607120124 27281131 PMC5427777

[B49] Primeaux S. Wilson S. McDonald A. Mascagni F. Wilson M. (2006). The role of delta opioid receptors in the anxiolytic actions of benzodiazepines. Pharmacol. Biochem. Behav. 85, 545–554. 10.1016/j.pbb.2006.09.025 17109943 PMC1892843

[B50] Ren Z.-Q. Zheng S.-Y. Sun Z. Luo Y. Wang Y.-T. Yi P. (2025). Resveratrol: molecular mechanisms, health benefits, and potential adverse effects. MedComm 6, e70252. 10.1002/mco2.70252 40502812 PMC12152427

[B51] Rezq S. Alsemeh A. E. D’Elia L. El-Shazly A. M. Monti D. M. Sobeh M. (2020). Thymus algeriensis and Thymus fontanesii exert neuroprotective effect against chronic constriction injury-induced neuropathic pain in rats. Sci. Rep. 10, 20559. 10.1038/s41598-020-77424-0 33239680 PMC7688974

[B52] Saadabadi A. Rantanen M. Marimuthu P. Koivisto A. Eklund P. C. Salo‐Ahen O. M. H. (2025). Insights into molecular interactions and biological effect of natural stilbenoids at the TRPA1 ion channel. ChemMedChem 20, e202400501. 10.1002/cmdc.202400501 39432420 PMC11793850

[B53] Safieh-Garabedian B. Nomikos M. Saadé N. (2019). Targeting inflammatory components in neuropathic pain: the analgesic effect of thymulin related peptide. Neurosci. Lett. 702, 61–65. 10.1016/j.neulet.2018.11.041 30503917

[B54] Shiao R. Lee-Kubli C. A. (2018). Neuropathic pain after spinal cord injury: challenges and research perspectives. Neurotherapeutics 15, 635–653. 10.1007/s13311-018-0633-4 29736857 PMC6095789

[B55] Sobeh M. Mahmoud M. F. Rezq S. Abdelfattah M. A. O. Mostafa I. Alsemeh A. E. (2020). Haematoxylon campechianum extract ameliorates neuropathic pain *via* inhibition of NF-κB/TNF-α/NOX/iNOS signalling pathway in a rat model of chronic constriction injury. Biomolecules 10, 386. 10.3390/biom10030386 32131490 PMC7175380

[B56] Sturman O. Germain P.-L. Bohacek J. (2018). Exploratory rearing: a context- and stress-sensitive behavior recorded in the open-field test. Stress 21, 443–452. 10.1080/10253890.2018.1438405 29451062

[B57] Suarez S. D. Gallup G. G. (1981). An ethological analysis of open-field behavior in rats and mice. Learn. Motiv. 12, 342–363. 10.1016/0023-9690(81)90013-8

[B58] Sun J. Zhang X. Broderick M. Fein H. (2003). Measurement of nitric oxide production in biological systems by using griess reaction assay. Sensors 3, 276–284. 10.3390/s30800276

[B59] Sun J. Li J.-Y. Zhang L.-Q. Li D.-Y. Wu J.-Y. Gao S.-J. (2021a). Nrf2 activation attenuates chronic constriction injury-induced neuropathic pain *via* induction of PGC-1α-Mediated mitochondrial biogenesis in the spinal cord. Oxid. Med. Cell. Longev. 2021, 9577874. 10.1155/2021/9577874 34721761 PMC8554522

[B60] Sun Z. Wang X. Xu Z. (2021b). SIRT1 provides new pharmacological targets for polydatin through its role as a metabolic sensor. Biomed. Pharmacother. 139, 111549. 10.1016/j.biopha.2021.111549 33901876

[B61] Szewczyk A. Jamroz-Wiśniewska A. Haratym N. Rejdak K. (2022). Neuropathic pain and chronic pain as an underestimated interdisciplinary problem. Int. J. Occup. Med. Environ. Health 35, 249–264. 10.13075/ijomeh.1896.01676 35040826 PMC10464730

[B62] Tan E. C. Bahrami S. Kozlov A. V. Kurvers H. A. J. M. Ter Laak H. J. Nohl H. (2009). The oxidative response in the chronic constriction injury model of neuropathic pain. J. Surg. Res. 152, 84–88. 10.1016/j.jss.2008.03.035 18708193

[B63] Vanderwall A. G. Milligan E. D. (2019). Cytokines in pain: harnessing endogenous anti-inflammatory signaling for improved pain management. Front. Immunol. 10, 3009. 10.3389/fimmu.2019.03009 31921220 PMC6935995

[B64] Wang J. Men Y. Wang Z. (2023). Polydatin alleviates chronic stress-induced depressive and anxiety-like behaviors in a mouse model. ACS Chem. Neurosci. 14, 977–987. 10.1021/acschemneuro.2c00758 36802487

[B65] Ye J. Piao H. Jiang J. Jin G. Zheng M. Yang J. (2017). Polydatin inhibits mast cell-mediated allergic inflammation by targeting PI3K/Akt, MAPK, NF-κB and Nrf2/HO-1 pathways. Sci. Rep. 7, 11895. 10.1038/s41598-017-12252-3 28928455 PMC5605538

[B66] Zamani K. Fakhri S. Kiani A. Abbaszadeh F. Farzaei M. H. (2025). Rutin engages opioid/benzodiazepine receptors towards anti-neuropathic potential in a rat model of chronic constriction injury: relevance to its antioxidant and anti-inflammatory effects. Naunyn. Schmiedeb. Arch. Pharmacol. 398, 9199–9213. 10.1007/s00210-025-03842-4 39912904

[B67] Zeilhofer H. U. Ralvenius W. T. Acuña M. A. (2015). Restoring the spinal pain gate: GABA(A) receptors as targets for novel analgesics. Adv. Pharmacol. 73, 71–96. 10.1016/bs.apha.2014.11.007 25637438

